# Estimating and evaluating the rice cluster distribution uniformity with UAV-based images

**DOI:** 10.1038/s41598-021-01044-5

**Published:** 2021-11-02

**Authors:** Xiaohui Wang, Qiyuan Tang, Zhaozhong Chen, Youyi Luo, Hongyu Fu, Xumeng Li

**Affiliations:** 1 College of Agriculture, Hunan Agricultural University, Changsha, 410128 People’s Republic of China; 2 College of Information and Intelligence Science, Hunan Agricultural University, Changsha, 410128 People’s Republic of China

**Keywords:** Computational biology and bioinformatics, Plant sciences

## Abstract

The uniformity of the rice cluster distribution in the field affects population quality and the precise management of pesticides and fertilizers. However, there is no appropriate technical system for estimating and evaluating the uniformity at present. For that reason, a method based on unmanned aerial vehicle (UAV images) is proposed to estimate and evaluate the uniformity in this present study. This method includes rice cluster recognition and location determination based on the RGB color characteristics of the seedlings of aerial images, region segmentation considering the rice clusters based on Voronoi Diagram, and uniformity index definition for evaluating the rice cluster distribution based on the variation coefficient. The results indicate the rice cluster recognition attains a high precision, with the precision, accuracy, recall, and F1-score of rice cluster recognition reaching > 95%, 97%, 97%, 95%, and 96%, respectively. The rice cluster location error is small and obeys the gamma (3.00, 0.54) distribution (mean error, 1.62 cm). The uniformity index is reasonable for evaluating the rice cluster distribution verified via simulation. As a whole process, the estimating method is sufficiently high accuracy with relative error less than 0.01% over the manual labeling method. Therefore, this method based on UAV images is feasible, convenient, technologically advanced, inexpensive, and highly precision for the estimation and evaluation of the rice cluster distribution uniformity. However, the evaluation application indicates that there is much room for improvement in terms of the uniformity of mechanized paddy field transplanting in South China.

## Introduction

The horizontal distribution of seedlings constitutes the basis for the formation of the spatial structure of rice population, and this affects the population development, canopy light distribution and radiation utilization of rice, thus influencing rice yields^1,2^. An excessive uneven distribution often results in yield reduction under throwing rice cultivation, as uneven seedling throwing causes local core grass formation, resulting in an uneven population distribution, which directly affects the field ventilation and light transmission and induces uneven fertilizer absorption and susceptibility to diseases and lodging^3^. However, the research on the effect of the seedling plane distribution on rice growth is insufficient^2^. The effect of the spatial distribution of rice clusters on rice production may become an important component of cultivation technology research^4,5^.

Artificial fixed-point throwing and fixed-point transplanting can control the uniformity of the seedling plane distribution to a certain extent, and avoid an excessively uneven seedling plane distribution to achieve the goal of a stable and high yield. However, artificial fixed-point throwing and fixed-point transplanting require much labor and a high labor intensity. Mechanical transplanting and throwing have been widely promoted in rice growing areas in southern China due to their advantages of a high transplanting efficiency and low labor intensity^4,5^. Therefore, it is particularly important to evaluate the evenness of the seedling plane distribution in a convenient and rapid way, and guide the improvement in machine performance and transplanting effect. By analyzing the existing literatures, we found that one of the difficulties involved the analysis of the plane area occupied by rice bushes. It is challenging to control and implement the plane distribution of seedlings when first planning the plane distribution and then arranging the rice clusters according to the determined planning points. Based on existing seedlings in the field, it is difficult to determine the plane area occupied by rice clusters through field measurements. Therefore, it is an important technical problem to obtain the information on the plane distribution of rice burrows quickly and accurately and to evaluate the uniformity of the rice burrow plane distribution. However, relevant studies are rare, which should be addressed.

The rice cluster position calibration issue is an important problem when evaluating the evenness of the seedling distribution. Unmanned aerial vehicle (UAV) remote sensing systems equipped with high-definition digital cameras provide the advantages of a low cost and high throughput, and are widely applied in image information acquisition^6–8^. Image target recognition is the key technology of information acquisition based on aerial images. In recent decades, the theory of image target recognition has been greatly developed. The first step of object recognition entails the extraction of to extract feature parameters. The commonly considered image features include color, texture, shape and spatial relationship features^9,10^. Image feature extraction methods can be divided into traditional methods and neural network-based techniques^11–13^. The second step of target recognition is to input the extracted features into a classifier for classification purposes. According to the feature classification algorithm, classification methods are divided into two categories: probability density-based methods^14^ and discriminant function-based methods^15^. A large number of studies has been carried out on the recognition of plants or organs by predecessors, which have been widely applied to agricultural intelligence, such as the classification, recognition and positioning of fruits such as apples and strawberries^16–19^.and determination of the tiller and panicle numbers of rice, wheat, maize and other crops^20–24^. Therefore, aerial images and target recognition are feasible techniques to accurately identify and locate rice clumps.

The determination of the spatial area occupied by rice clusters is another important problem in the evaluating the evenness of seedling distribution evenness. Dai^1,2^ made efforts to determine the hole area via Voronoi Diagram, and the area occupied by the rice plants in each hole was a convex polygon. The relationship between the occupied area and shape parameters of each hole and the number of panicles per hole, biological yield and grain weight was studied. However, the rationality of the uniformity determination method was not been verified.

The technical bottlenecks of rice crop research and production evaluation include the acquisition of information on the rice burrow plane distribution and the evaluation of the uniformity level of rice burrow plane distribution under nonuniform conditions. To solve these technical problems, this paper explores and tests a technical system for the acquisition of information on the rice burrows plane distribution and evaluating of the uniformity of the rice burrows plane distribution based on UAV aerial images. The location of rice clusters is identified and demarcated according to the color and shape features of UAV images captured during the rice greening period. Voronoi Diagram is applied to determine the area of the space occupied by rice clumps. The uniformity of the rice cluster distribution is determined according to the variation in the rice cluster area.

## Materials and methods

### Image acquisition and experimental design

The field experiment was conducted at the Hongshuo Rice Planting Cooperative in the Datong Lake District (N 29° 10′ 17″, E 112° 28′ 45″), Yiyang city, Hunan Province. This area experiences a subtropical monsoon humid climate, with abundant rainfall and sufficient light and heat. The area is an important national rice planting and *commercial grain production base*. *The flat terrain* characteristics of the field provides favorable conditions for *drone aerial photography*. The study selected an experimental field in the demonstration area supported by the *Earmarked Fund for China Agriculture Research System*. The experimental field within the demonstration area includes three transplanting modes: *mechanical transplanting, mechanical seedling throwing, and manual seedling throwing*. A *quad-rotor drone* (*Cv. DJI Phantom 4*) was employed to capture field images of the *demonstration area* on 6 d after transplanting (April—May 2019). The UAV exhibits a total mass of 1391 g, a maximum flight time of 30 min, and a *1-inch CMOS image sensor*, with effective pixels up to 20.8 million, and a maximum resolution of 5472 × 3648. To avoid the influence of notable light, periods with sunny and windless weather conditions were selected for *aerial photography*. Aerial photography was performed from 8:00 to 9:00 am Beijing Time. Good light conditions facilitated the subsequent *image processing*. The aerial photography height was 6 m above the ground, the *gimbal pitch angle* was -90°, and the aerial images were saved in the JPG format. The camera parameters were set before the aerial photography mission, the *automatic* exposure mode of the digital camera was selected *to be automatic*, and the UAV was stabilized to perform the photo shot mode. A total of 160 images was collected, which included the distributions of seedlings under all three transplanting modes mentioned above.

### Cluster recognition and location determination

After rice transplanting, the seedlings first withered, with the color of the seedlings changing from green to yellow, and thereafter were revived, with the color of the seedlings changing from yellow to green. The seedlings remained upright in 5–8 d after transplanting and exhibited a relatively *consistent color of light-yellow color*. This period provided the best opportunity to distinguish the transplanted seedlings from the environmental background such as the soil, based on the *color space*. This article mainly includes the following steps to identify and determine the location of seedlings.

**Figure 1:**
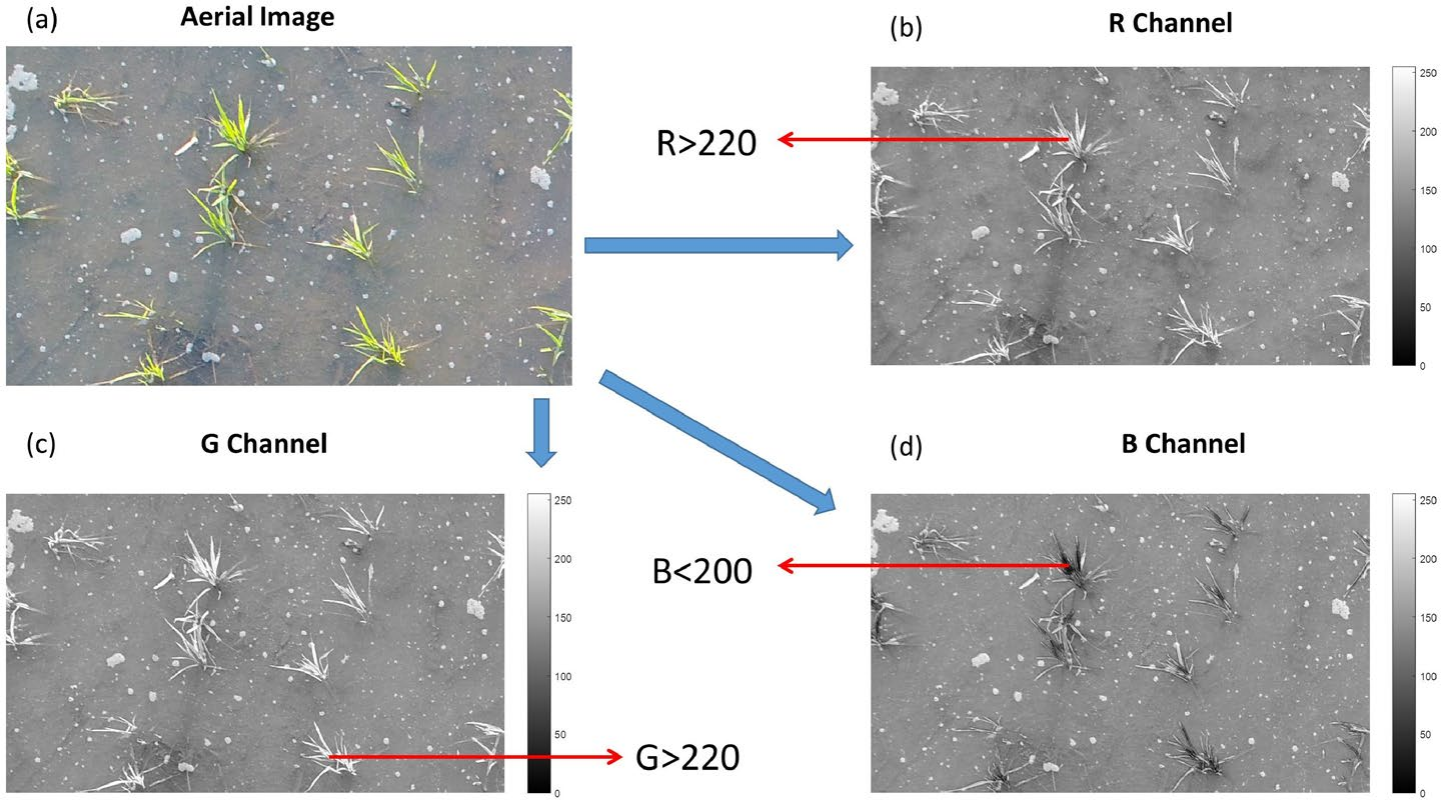
RGB color characteristics of the seedlings.

1. *Image binarization* was performed according to the RGB color characteristics of the seedlings. *The R and G channel values of the seedlings and* Bubble were much higher than those of the soil background, and the Bubble B channel value was generally higher than that of the seedlings. Therefore, mean R and G channel > 220, and a mean B channel value < 200 considered in image binarization (Eq. 1, Fig. 1).

1 $${\text{f}}\left( {{\text{x}},{\text{y}}} \right) = \left\{ {\begin{array}{*{20}l} 1 \hfill & {\frac{{R\left( {x,y} \right)}}{2} + \frac{{G\left( {x,y} \right)}}{2} > 220,\;\;B\left( {x,y} \right) < 200} \hfill \\ 0 \hfill & {other} \hfill \\ \end{array} } \right.$$

where (x,y) is the pixel location, R(x,y) is the red channel, G(x,y) is the green channel, and B(x,y) is the blue channel. The pixel value of binary image pixel location (x,y) is denoted as f(x,y).

2. *Noise removal* The *connected components* of a given binary image were labeled to calculate the main axis length of each component, and any the component with a main axis length smaller than 3 was eliminated to remove noise from the image^25^.

**Figure 2:**
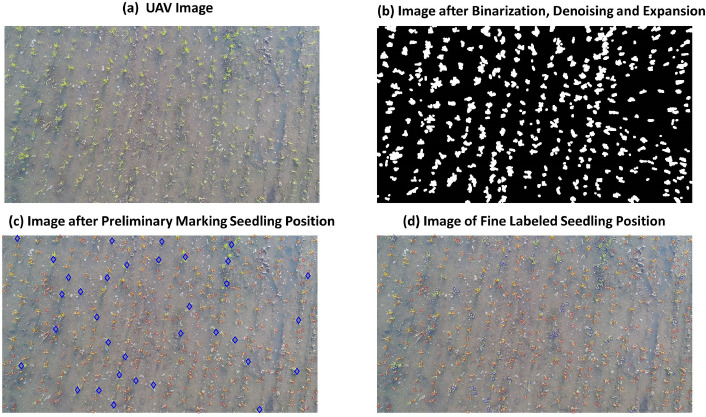
Rice cluster recognition and location labeling process. (**a**): UAV image; (**b**): Image afer binarization, denosing and expansion; (**c**): Image afer preliminary marking seedling positon, the blue diamond indicates the larger component within the > 85th quantile and the red dots indicate the initially labeled seedling positions; (**d**): Image of fine labeled seedling position, the red and blue dots denote the preliminary and detailed labeled seedling positions, respectively.

The position of the seedlings was labeled. A *disk-shaped structuring element* with a *pixel* radius *of 11* was employed in the *dilation operation*^26^ to connect rice clusters originating from the same root hole. The connected *components* were preliminarily labeled, thereby arranging the areas in descending order. The components within the 85th quantile were identified as rice clusters, and the center of gravity of each component was labeled as the position of the rice clusters. Regarding the remaining *components*, a *disk-shaped structuring element comprising 5 pixels* was applied in the *corrosion operation* to label the *connected components* of the *corrosion image*. Areas containing more than 400 pixels with a major axis length larger than 40 pixels were considered as rice clusters, and the position of the *center of gravity* was regarded as the position of the rice cluster for detailed labeling purposes (Fig. 2).

### Region segmentation based on Voronoi Diagram

Adopting the rice clusters as the basic unit, the component assigned to the rice clusters via Voronoi Diagram should match the four criteria. ① Each cluster of rice plants is located within a unique component (i.e., uniqueness). ② The components occupied by any two rice plant clusters should not overlap (i.e., mutual exclusion). ③ The component occupied by all the rice clusters should exactly cover the entire component (i.e., the principle of full coverage); ④ The region occupied by two rice clusters should resemble a convex polygon, and its boundary should pass the midpoint of the line connecting the two rice clusters (i.e., the principle of space sharing).

**Figure 3:**
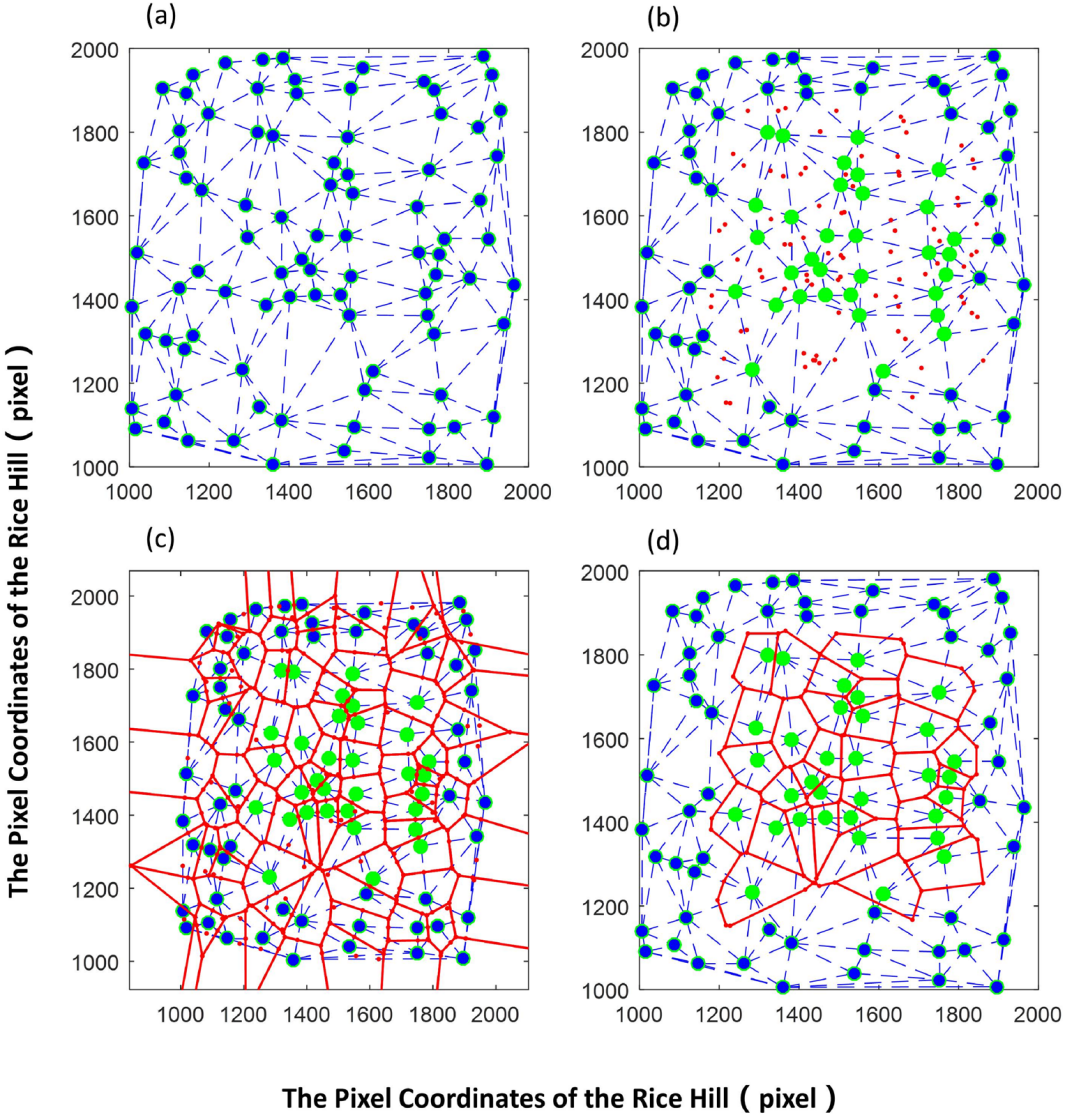
Segmentation process of the region occupied by the rice clusters. (**a**): Segment DT map; (**b**): Segment DT map with part of the outer center of the triangle; (**c**): Segment DT map of the region occupied by rice clusters (full image); (**d**): Segment DT map of the region occupied by the rice clusters (partial image). Large dot—rice cluster; red dot—outer center of the triangle; blue dotted line—edge of the triangle; red solid—borderline of the region occupied by the rice clusters.

Voronoi Diagram for determining the rice clusters occupied region (Fig. 3) was established with the following steps. (1) Delaunay triangulation (DT)^27^ was performed according to Lawson's algorithm, with the DT function applied in MATLAB 2016a. (2) The polygon of the area occupied by the rice clusters was individually solved. The solution steps are as follows: ① In the DT segmentation diagram, the Delaunay triangle with the rice cluster as the vertex was identified, with the outer center of each Delaunay triangle determined accordingly. ② The outer centers of all triangles were connected in sequence in the clockwise direction to obtain the polygonal area that includes the rice clusters.

### Evaluation indicators of the recognition precision

In this paper, four indicators of the precision, accuracy, recall, and F1-score were adopted to evaluate the *precision* of rice cluster recognition^28^.

2 $${\text{Precision}} = {\text{TP/}}\left( {{\text{TP}} + {\text{FP}}} \right)$$

3 $${\text{Accuracy}} = \left( {{\text{TP}} + {\text{FN}}} \right){/}\left( {{\text{TP}} + {\text{FP}} + {\text{TN}} + {\text{FN}}} \right)$$

4 $${\text{Recall}} = {\text{TP/}}\left( {{\text{TP}} + {\text{FN}}} \right)$$

5 $${\text{F}}1 = 2{\text{Precision}} \times {\text{Recall/}}\left( {{\text{Precision}} + {\text{Recall}}} \right)$$

where TP denotes the number of rice clusters correctly identified, TN denotes the number of non-rice clusters correctly identified (TN = 0 in this article), FP denotes the number of non-rice clusters incorrectly identified as rice clusters, and FN denotes the number of unidentified rice clusters.

### Uniformity index of the rice cluster distribution

To eliminate the influence of the rice cluster density, this paper adopted the coefficient of variation (CV) as a heterogeneity index for evaluating the heterogeneity in the rice cluster distribution in the region of interest^29^.

6 $${\text{CV}} =\upsigma {/}{\overline{\text{x}}}$$

7 $$\upsigma = \sqrt {\mathop \sum \limits_{i = 1}^{n} \left( {x_{i} - \overline{x}} \right)^{2} {/}n}$$

8 $$\overline{x} = \mathop \sum \limits_{i = 1}^{n} x_{i} {/}n$$

where $x_{i}$ is the area of the region occupied by the rice clusters,$n$ is the number of rice clusters, $\overline{x}$ is the mean. and σ is the variance. The larger the heterogeneity index value is, the more uneven the rice clusters distribution. Accordingly, the uniformity index is defined as the reciprocal of CV, the larger the uniformity index value is, the more even the rice clusters distribution.

## Results and analysis

### Precision of rice cluster recognition

Six images of seedlings captured by drones were randomly selected, and the rice clusters were identified according to the rice cluster recognition process above. As shown in Fig. 2, TP, FP and FN were artificially counted. Based on Eqs. (2)–(5), precision evaluation metrics of preciseness in rice cluster recognition were computed, such as Precision, accuracy, recall, and F1-score (Table 1).

**Table 1:** Precision of rice cluster recognition.

Image sample	S1	S2	S3	S4	S5	S6	Image sample	S1	S2	S3	S4	S5	S6	Mean	Variance
TP	210	244	250	233	267	233	Precision	0.95	0.98	0.97	0.97	0.97	0.96	0.97	0.01
FN	16	8	13	9	12	11	Accuracy	0.96	0.98	0.97	0.98	0.98	0.96	0.97	0.01
FP	10	4	9	6	7	9	Recall	0.93	0.97	0.95	0.96	0.96	0.95	0.95	0.01
TN	0	0	0	0	0	0	F1	0.94	0.98	0.96	0.97	0.97	0.96	0.96	0.01

### Errors of location determination

**Figure 4:**
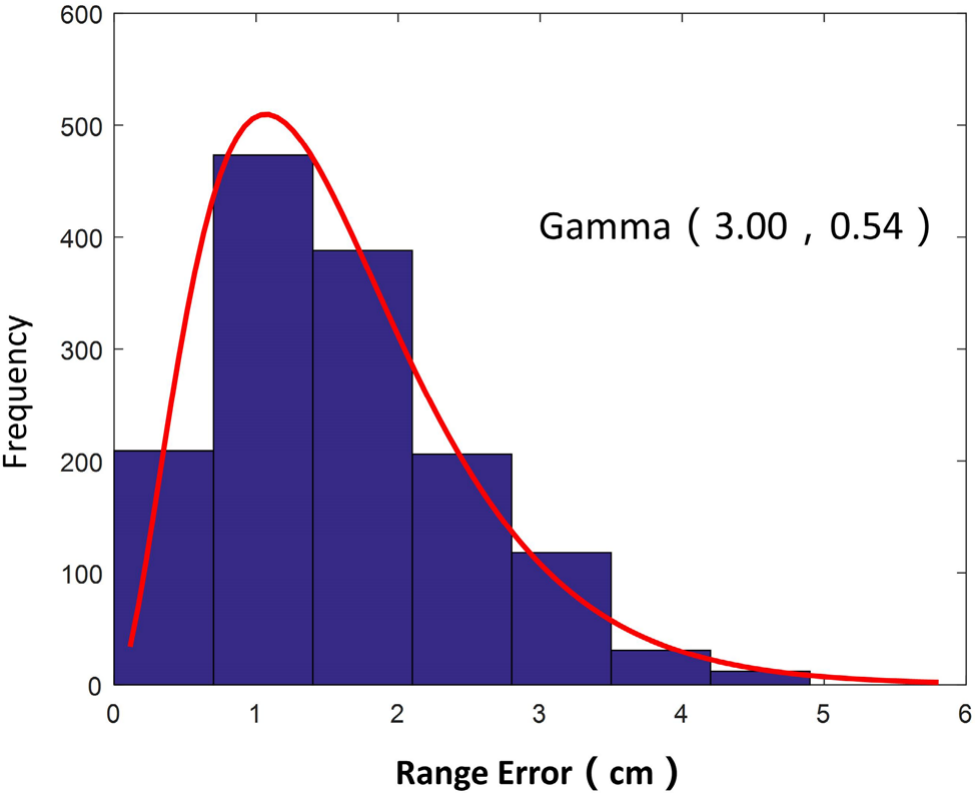
Errors in the position distribution of the rice clusters.

Based on the six selected UAV-based images of field seedlings in the field, 1473 rice clusters were correctly recognized in the rice cluster recognition process, with the determined location. Moreover, the positions of these 1473 rice clusters were correctly labeled manually. *The pixel distance* of the same rice cluster position calibrated by the above two methods was computed, and converted into the *actual distance* according to the ratio of the *pixel scale* to the *actual scale*. The rice cluster location error was defined as that actual distance for each one. The rice cluster location error distribution obeys the gamma (3.00, 0.54) distribution with 1.62 cm of mean error, shown in Fig. 4.

### Homogeneity index of the simulated rice cluster distributions

Based on stochastic simulation, the rationality of the evaluation metrics of the rice cluster distribution was verified. Three methods were designed to randomly simulate the rice clusters distribution. ① Considering the lattice distribution, the positions of the rice clusters were randomly perturbed obeying a normal distribution with the fixed expectation of zero but variable variance levels (perturbation variance). ② Based on the lattice distribution, rice clusters were randomly omitted at variable ratios (omission ratio); ③ A two-dimensional uniform random distribution was applied to simulate a completely random distribution. Evaluation metrics of the heterogeneity in the rice cluster distribution were computed to analyze the relationship between the evaluation metrics and the randomness.

#### Simulation of the rice clusters distribution with positions perturbation

**Figure 5:**
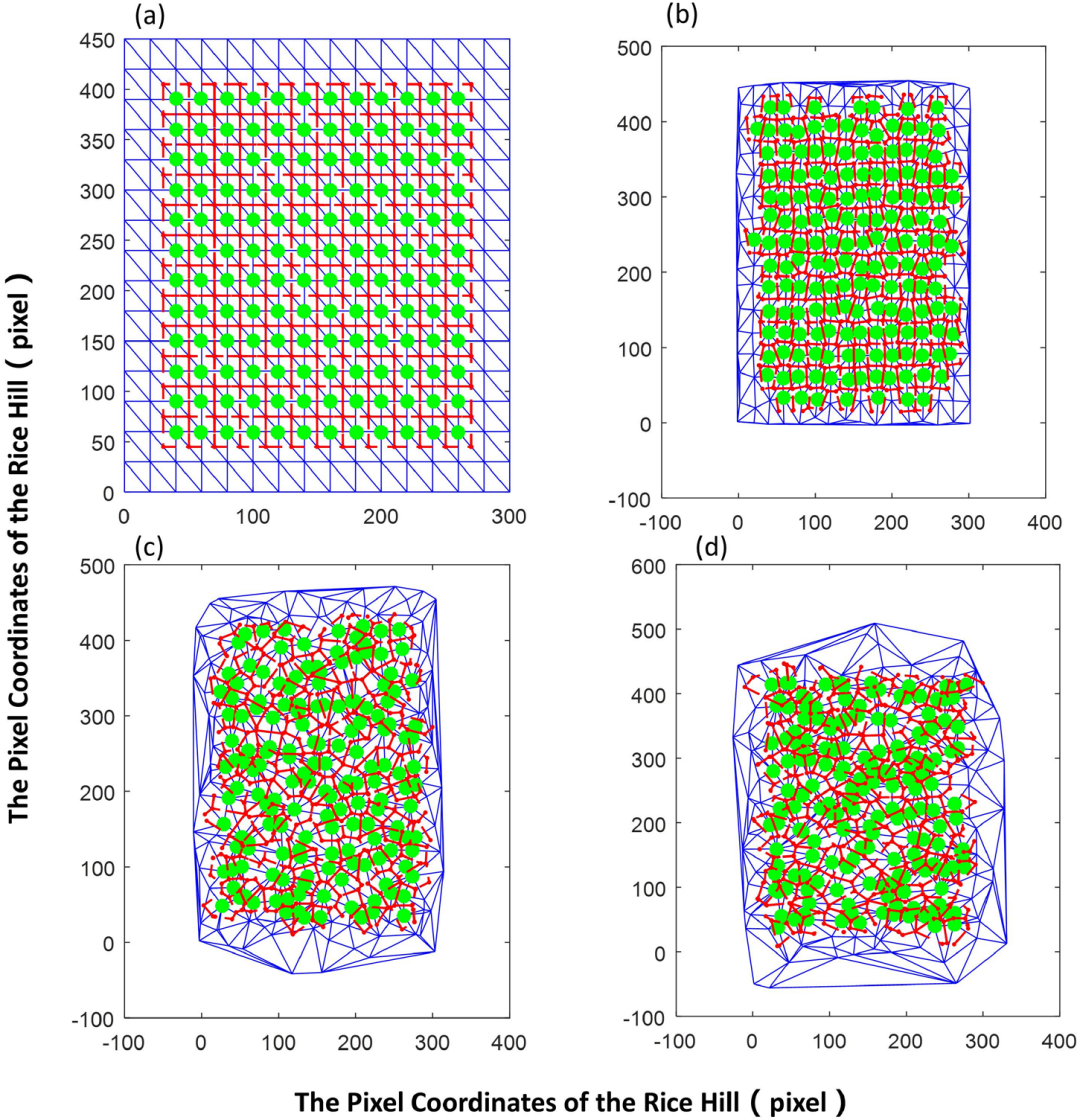
Distribution of the rice clusters randomly simulated at different variance levels of random perturbation and the region occupied by the rice clusters. Subfigures (**a**–**d**) show σ = 0RD, 0.1RD, 0.6RD, and RD, respectively. Large dot, the position of the rice cluster; red dot, the outer center of the triangle; blue dotted line, the triangular segmentation result; red solid line, the boundary line of the region occupied by the rice cluster.

The row spacing ratio was set to 2:3, with a field size of 15 × 15 clusters per plot. The position of the rice clusters was simulated with a variable describing a random disturbance under the standard lattice layout. Equation (9) was applied to simulate the rice cluster position distributions (Fig. 5).

9 $$\left\{ {\begin{array}{*{20}l} \begin{gathered} x\left( {i,j} \right) = PD \times i + N\left( {0,\sigma_{1}^{2} } \right) \hfill \\ y\left( {i,j} \right) = RD \times j + N\left( {0,\sigma_{2}^{2} } \right) \hfill \\ \end{gathered} \hfill & \begin{gathered} i,j = 1,2, \ldots ,n; \hfill \\ \sigma_{1} = 0,0.1PD,0.2PD, \ldots ,PD \hfill \\ \sigma_{2} = 0,0.1RD,0.2RD, \ldots ,RD; \hfill \\ \end{gathered} \hfill \\ \end{array} } \right.$$

In the above equation, (i, j) denotes the coordinates (row, column) of the rice clusters. PD denotes the plant spacing, RD denotes the row spacing, and $N\left( {0,\sigma^{2} } \right)$ is the normal distribution. The randomness of the rice cluster distribution was adjusted according to the value of variance value (σ^2^), with σ = 0 indicating the standard lattice distribution. The larger the σ value is, the higher the randomness of the rice cluster distribution.

#### Simulation of the rice cluster distribution with cluster omission

**Figure 6:**
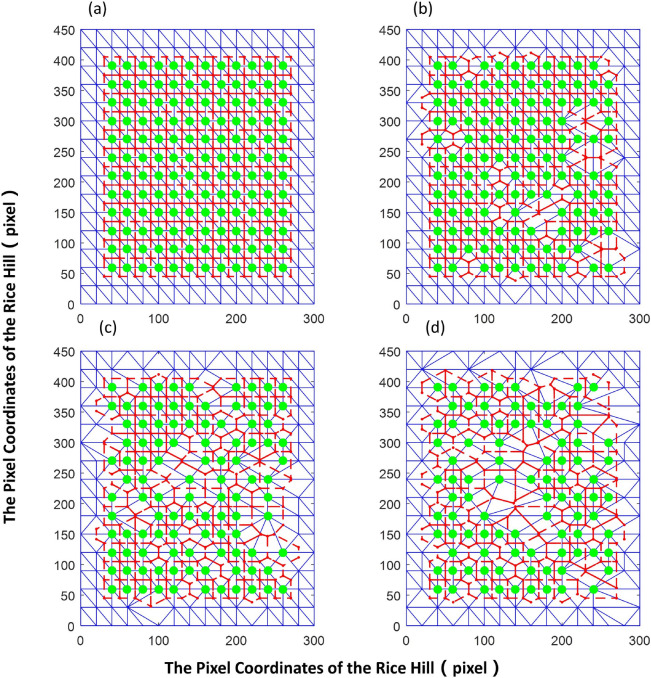
Distribution of the rice clusters randomly simulated at the different omission ratio and the regions occupied by the rice clusters. (**a**–**d**) show omission ratios of 0%, 10%, 20%, and 50%, respectively. The large dots indicate the positions of the rice cluster; the red dots indicate the outer centers of the triangles; the blue dotted line indicates the triangular segmentation result; and the red solid line indicates the boundary line of the region occupied by the rice cluster.

The row spacing ratio was set to RD: PD = 2:3, with a field size of 15 × 15 clusters per plot. The probability of missing a given rice cluster was assumed to be the same, which was simulated with a uniform distribution. Maintaining the boundary of the rice clusters, a 14 × 14 clusters was generated randomly, with the rice cluster omissions levels determined according to the probability *P* (*P* = 1%, 2%, …, 50%) (Fig. 6).

The area of the region occupied by each rice cluster was computed. Evaluation metrics of the rice cluster distribution were calculated accordingly (Eqs. 6–8), and then the relationship between the evaluation metrics and randomness was analyzed as shown in Figs. 5 and 6.

**Figure 7:**
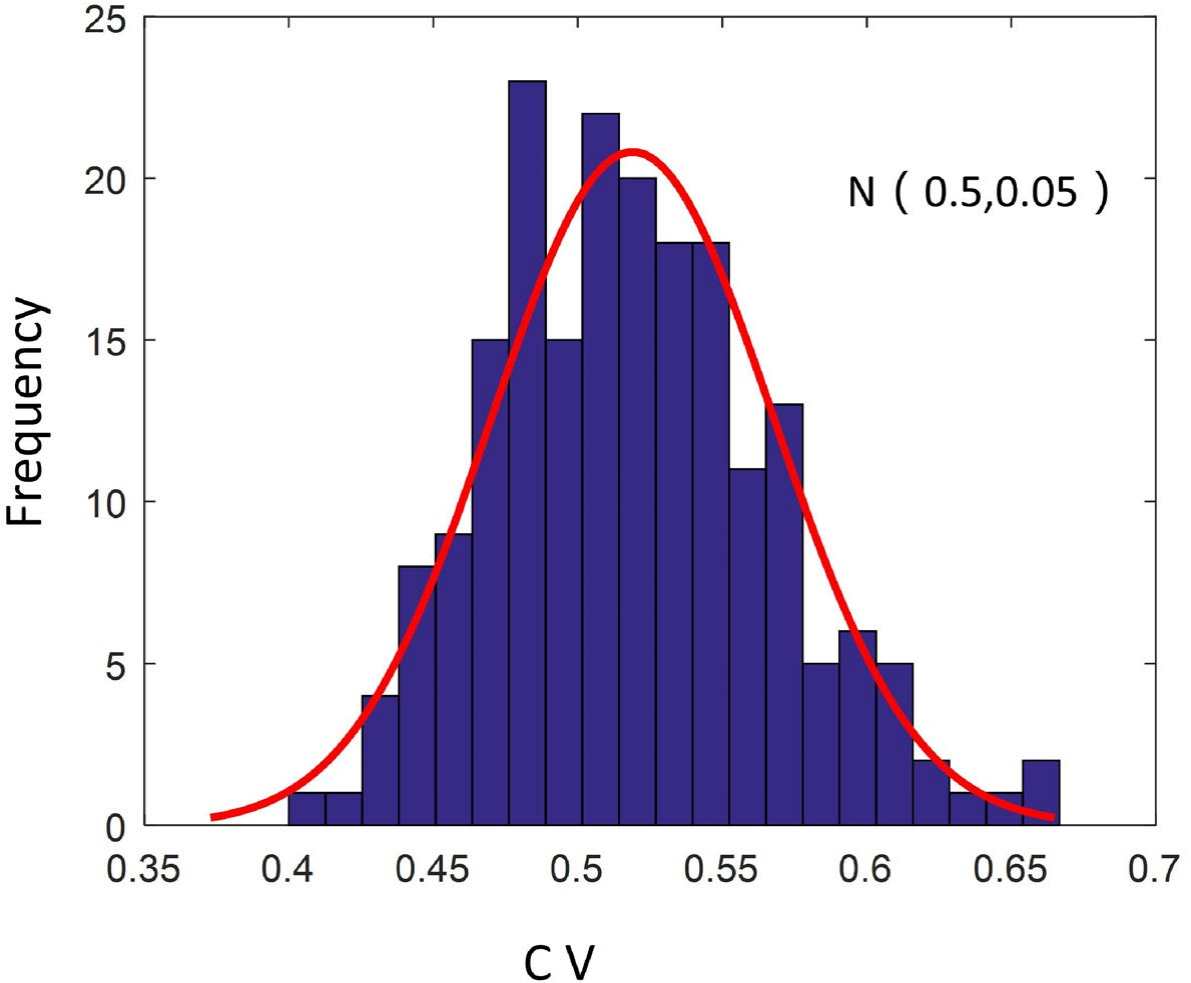
Histogram of heterogeneity index CV for the randomly simulated rice cluster distributions.

**Figure 8:**
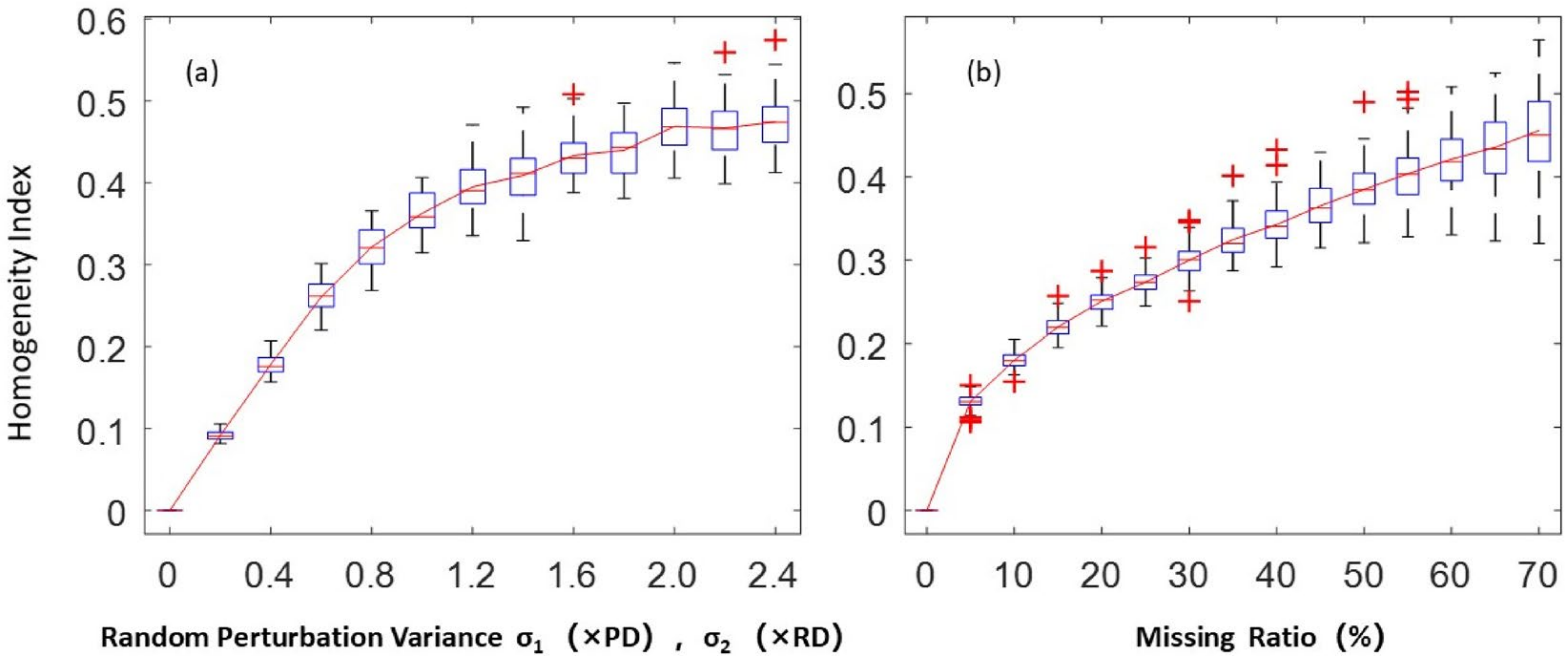
Trend of the changes in the heterogeneity index with the increasing variance under random perturbation (**a**) and with the omission ratio (**b**).

The results suggested that: (1) the heterogeneity index of the rice cluster lattice distribution was 0, suggesting a strictly uniform distribution. (2) The heterogeneity index of the fully randomly distributed rice clusters obeyed a normal distribution with a mean value of 0.5 and a variance of 0.05 (Fig. 7). (3) The higher the degree of random position perturbation, the higher the nonuniformity was (Fig. 8a). (4) The greater the proportion of random omissions, the higher the heterogeneity index of the rice cluster distribution was, but with a decelerating increase rate was observed (Fig. 8b).

### Precision of heterogeneity index calculation

**Figure 9:**
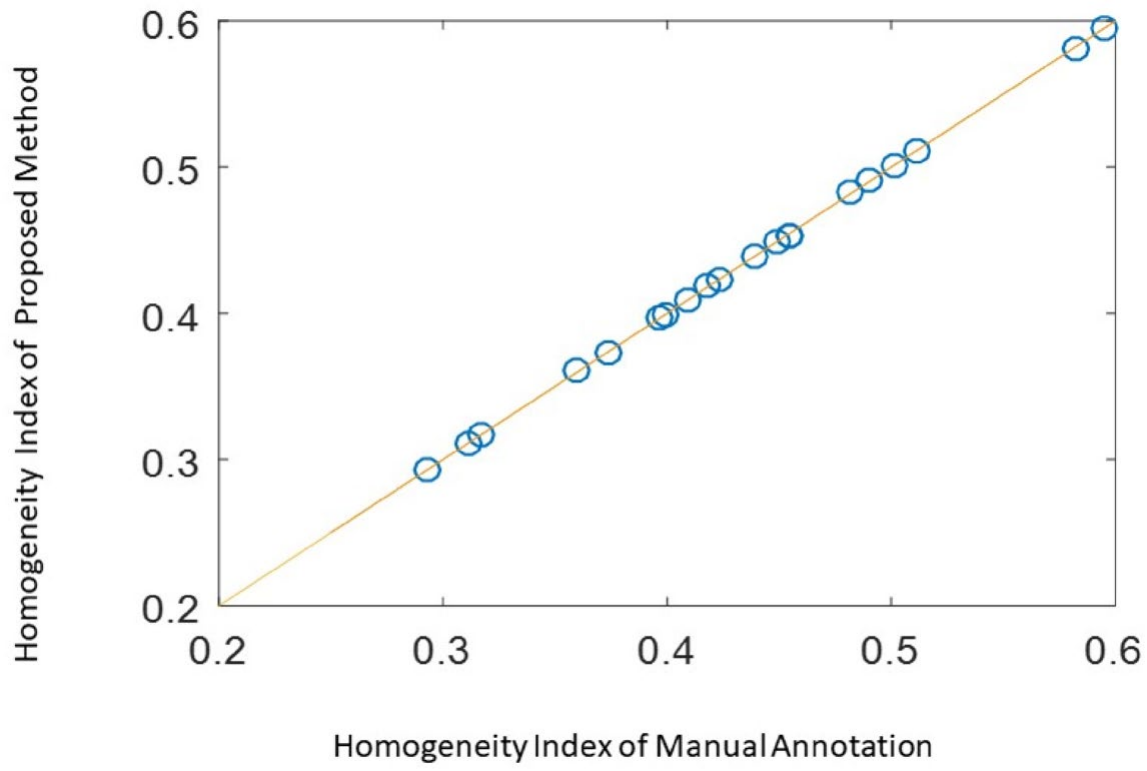
Validity evaluation of the method to calculate the distribution quality index of the rice clusters.

Based on 20 UAV images, The proposed method and manual method calibrated the position of rice cluster and then calculate the heterogeneity index of rice cluster distribution in the field. The result indicated the relative error of the heterogeneity index value calculated by the two methods is less than 0.01% (Fig. 9). Therefore, the two methods to calculate the heterogeneity index are approximately equivalent.

### Heterogeneity index under the different transplanting modes

**Figure 10:**
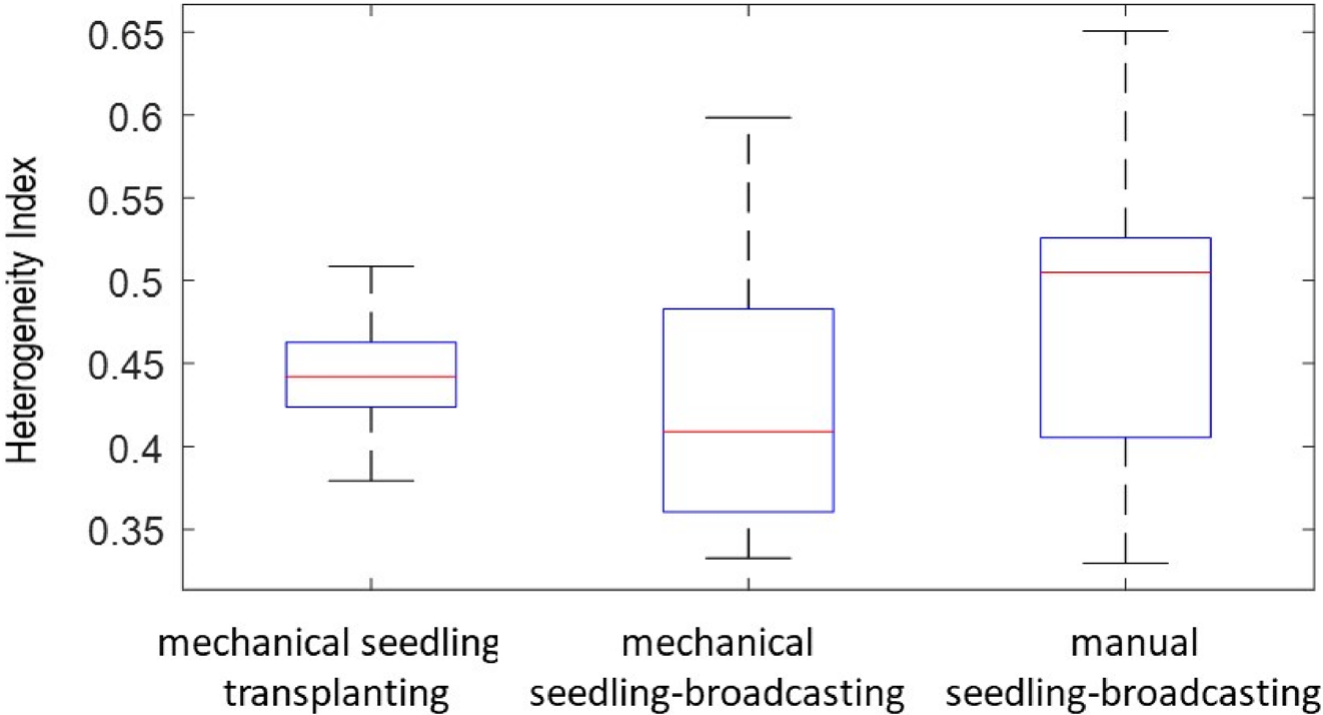
Comparison of the distribution quality of rice clusters under three transplanting ways.

As shown in Fig. 10, the evaluation index of the distribution quality was computed from 45 UAV photos for each of the three transplanting modes i.e., mechanical seedling transplanting, mechanical seedling throwing, and manual seedling throwing. The results indicated that the rice clusters distribution in actual production was heterogeneous, and the area of the region occupied by the rice clusters varied greatly with the heterogeneity index value in ascending order following mechanical seedling throwing < mechanical seedling transplanting < manual seedling throwing.

## Discussion

### Process evaluation of the method

This paper provides a method to evaluate the rice clusters distribution quality based on UAV-based aerial images. The evaluation processes mainly include (1) the acquisition of aerial images; (2) rice cluster recognition and rice cluster position determination based on the RGB color characteristics of the rice clusters, and (3) calculation of the area of the region occupied by the rice clusters with the *triangulation algorithm*. (4) CV, i.e., the evaluation index of the rice clusters distribution quality, was determined based on the area of the region occupied by the rice clusters.

The evaluation method of the rice cluster distribution quality of in this paper is scientific, reasonable, feasible, convenient, technologically advanced, and inexpensive. Specifically, (1) based on UAV-based aerial images, the data acquisition process is convenient and inexpensive. (2) The definition of the evaluation index of the rice distribution quality is reasonable and scientific. (3) The computational method for the evaluation index of the rice distribution quality based on image processing technology and the triangulation algorithm is feasible under the given temporal and spatial complexity conditions and achieves a certain technological advancement.

Rice cluster recognition and location determination comprise the basis for the estimation method of the rice-cluster distribution quality. The main techniques include binarization, denoising, and dilation. The effect of image binarization depends on the color characteristics of the seedlings relative to the background color, i.e., the value and composition of the RGB color channels. The denoising effect depends on the noise magnitude. The dilation effect depends on the dilation degree, namely, the radius of the structuring elements applied in the dilation operations. In this paper, the seedlings attained an upright position 5–8 d after transplanting, with a consistently light-yellow color. The expected effect was obtained by adjusting the parameters mentioned above. However, the optimization process of the parameters depended on the time, altitude, light environment, farmland environment and other factors of aerial photography. In particular, to address complex field conditions, rice cluster recognition and location determination methods require further development to allow universal application.

### Precision evaluation of the method

This article mainly provides methods to identify and determine rice clusters, and evaluate the quality of rice cluster distribution. The evaluation results of the method effectiveness exhibit the following characteristics. (1) Rice cluster recognition achieves a high precision. The precision, accuracy, recall, and F1-score values of rice cluster recognition are > 95%, 97%, 97%, 95%, and 96%, respectively. (2) The error in the rice cluster position is small, with the error obeying the gamma (3.00, 0.54) distribution (mean error, 1.62 cm). (3) The heterogeneity index for evaluating the heterogeneity in the rice cluster distributions are reasonable. The results of the simulated rice clusters distributions under random perturbations and with random omissions conform to the common heterogeneity evaluation requirements. (4) The evaluation precision of the uniformity of the rice cluster distribution is high. This paper compares the spatial distribution quality evaluation method based on the UAV-based image recognition and the manual labeling method of the position of the rice clusters, with obtaining a relative error < 0.01%.

### Uniformity evaluation in actual production condition

Practically, the rice clusters distribution is heterogeneous, and the size of the area occupied by rice clusters varies greatly. The simulation results suggested that, compared to the *lattice distribution*, the heterogeneity in the rice cluster distribution is related to the random perturbation and omission levels of the rice clusters. The greater the random perturbation in the position and the omission proportion, the higher the distribution heterogeneity was. The comparison of the uniformity of the three transplanting ways suggested that the popularization of mechanization has effectively improved the uniformity of the rice clusters distribution in the field. However, the uniformity of the mechanized rice clusters distributions is similar to those simulated under random positional perturbations with 1.2 times row spacing variance or those simulated under random omissions at 40% omission ratios. Therefore, there remains still much room for the improvement in terms of the uniformity of the rice cluster distribution by mechanical transplanting in actual production.

Under ideal circumstances, i.e., an ideal mechanical walking route without any omission, the uniformity of the rice cluster distribution under the three transplanting methods theoretically follows mechanical seedling transplanting > mechanical seedling throwing > manual seedling throwing (in descending order). However, the actual field data in this paper suggested that the three transplanting methods achieved similar effects and practically followed mechanical seedling throwing > mechanical transplanting > manual seedling throwing (in descending order). this was likely attributable to irregular and uneven mechanical walking trajectories and speeds. Therefore, to improve the uniformity of the rice cluster distribution, the quality of machine operators, machine control performance, and seedling quality should be improved.

### Potential application

With increasing randomness of the rice clusters distribution in the field, the heterogeneity accordingly increases. This could increase the difficulty of precision planting and compromise the pesticide and fertilizer use efficiency levels and their application effects, sequentially affecting the quality of the rice population and thus the yield. *Perturbations and omissions* of rice cluster positions could increase the distribution heterogeneity, which is affected by the mechanical performance, the level and status of the machine operators, and the quality of seedlings. Therefore, the rice distribution quality evaluation method can also potentially assess the performance of seedling transplanting machines, the agronomic effect of seedling transplanting, and the effect of seedling transplanting operations. In addition, the quantification of the size of the area occupied by the rice clusters provides a technical support for research on the population quality of rice clusters under heterogeneous distribution conditions.

## Conclusion

In the present study, a uniformity evaluation method is established, which is verified to achieve a high precision. Moreover, compared to manual data acquisition, the UAV image-based method is feasible, convenient, technologically advanced and inexpensive. Hence, this method is easy to widely apply. However, the method application reveals that there is much room for improvement terms of the uniformity of mechanized paddy field transplanting in south China.
